# Second-Hand Tobacco Smoke Exposure: Results of Particulate Matter (PM_2.5_) Measurements at Hospitality Venues in Addis Ababa, Ethiopia

**DOI:** 10.3390/ijerph21081011

**Published:** 2024-07-31

**Authors:** Selamawit Hirpa, Noreen Dadirai Mdege, Terefe Gelibo Argefa, Yifokire Tefera, Selam Abraham Kassa, Winnie Awuor, Wakgari Deressa

**Affiliations:** 1 Department of Preventive Medicine, School of Public Health, Addis Ababa University, Addis Ababa P.O. Box 9086, Ethiopia; yifoomitu@yahoo.com (Y.T.); wakgari.deressa@aau.edu.et (W.D.); 2 Department of Health Sciences, University of York, York YO10 5DD, UK; noreen.mdege@york.ac.uk; 3 Development Gateway: An IREX Venture, 1100 13th St NW, Suite 800, Washington, DC 20005, USA; tagerfa@developmentgateway.org (T.G.A.); skassa@developmentgateway.org (S.A.K.); wawuor@developmentgateway.org (W.A.); 4 Centre for Research in Health and Development, York YO422BS, UK; 5 ICAP, Columbia University Mailman School of Public Health, Kirkos Sub-City, Addis Ababa P.O. Box 5566, Ethiopia

**Keywords:** second-hand smoke exposure, PM_2.5_, hospitality venues

## Abstract

Introduction: In Ethiopia, a comprehensive smoke-free law that bans smoking in all public areas has been implemented since 2019. This study aimed to evaluate compliance with these laws by measuring the air quality and conducting covert observations at 154 hospitality venues (HVs) in Addis Ababa. Methods: Indoor air quality was measured using Dylos air quality monitors during the peak hours of the venues, with concentrations of particulate matter <2.5 microns in diameter (PM_2.5_) used as a marker of second-hand tobacco smoke. A standardized checklist was used to assess compliance with smoke-free laws during the same peak hours. The average PM_2.5_ concentrations were classified as good, moderate, unhealthy for sensitive groups, unhealthy for all, or hazardous using the World Health Organization’s (WHO) standard air quality index breakpoints. Results: Only 23.6% of the venues complied with all smoke-free laws indicators. Additionally, cigarette and shisha smoking were observed at the HVs. Overall, 63.9% (95% confidence interval: 56–72%) of the HVs had PM_2.5_ concentrations greater than 15 µg/m^3^. The presence of more than one cigarette smoker in the venue, observing shisha equipment in the indoor space, and the sale of tobacco products in the indoor space were significantly associated with higher median PM_2.5_ concentration levels (*p* < 0.005). Hazardous level of PM_2.5_ concentrations—100 times greater than the WHO standard—were recorded at HVs where several people were smoking shisha and cigarettes. Conclusions: Most HVs had PM_2.5_ concentrations that exceeded the WHO average air quality standard. Stricter enforcement of smoke-free laws is necessary, particularly for bars and nightclubs/lounges.

## 1. Background

Second-hand tobacco smoke (SHS) exposes individuals to toxic and carcinogenic components [1]. Particulate matter (PM_2.5_) is a mixture of solid and liquid particles with a diameter of <2.5 µm, serving as a biomarker for SHS exposure in both indoor and outdoor public places [1,2,3,4,5]. Even short-term exposure to these particles has been linked to increased mortality due to cardiovascular, respiratory, and cerebrovascular diseases [4,6]. Studies measuring airborne nicotine concentrations [7] and PM_2.5_ concentrations [8] have reported higher SHS concentrations in hospitality venues (HVs) than in other public places. Tobacco smoking is the main source of PM_2.5_ pollution in indoor spaces, producing far more fine particles than other sources [5]. In Scotland, a study reported a significant reduction in PM_2.5_ concentration following a ban on smoking in pubs [9].

Article 8 of the World Health Organization (WHO) Framework Convention on Tobacco Control (FCTC) calls upon all parties to implement measures to safeguard the public from SHS exposure in indoor public spaces [10]. Ethiopia enacted a comprehensive tobacco control law in 2019 (Proclamation 1112/2019) [11] to create smoke-free public spaces, including HVs. The aim of such laws is to reduce SHS exposure and, consequently, protect the health of nonsmokers. Studies have shown that comprehensive smoke-free laws can effectively reduce SHS in public venues, increase the demand for smoking cessation, and decrease smoking among young people [12,13].

Studies conducted in African countries have shown a lack of knowledge and low compliance with smoke-free policies in various hospitality venues, such as hotels, bars, nightclubs, pubs, and restaurants [10,14]. A 2016 survey conducted in Ethiopia revealed that 60% of adults were exposed to second-hand smoke in bars and nightclubs and 31% in restaurants [15]. To evaluate the effective implementation of these laws, it is crucial to conduct observations and measure air quality in these establishments. In this study, the researchers used a low-cost air quality monitor, the Dylos1700 (Dylos, Riverside, CA, USA), to measure mass concentrations of PM with sizes ranging from 0.5 µm to 2.5 µm in HVs in Addis Ababa [5,16]. The primary objective of this study was to measure the mass concentration of particles within this range, which is indicative of exposure to second-hand smoke. The findings of this study will be useful for decision making regarding the implementation and enforcement of smoke-free laws in these venues in Ethiopia.

## 2. Methods

### 2.1. Study Area and Design

This cross-sectional study was conducted in Addis Ababa, Ethiopia, as part of a larger national study assessing compliance with smoke-free laws. The city administration consists of 11 sub-cities [17] housing nearly five million people [17]. Each sub-city is further divided into woredas, which represent the lowest public administration structure in Ethiopia.

### 2.2. Sample Size Estimation

The national study had a total sample size of 1300 HVs across 10 major cities in Ethiopia. This sample size was based on a similar study conducted in Uganda, which found that 82% of the surveyed HVs complied with ‘no active smoking’ in venues [18], with a 95% confidence level, 3% margin of error, a design effect of two, and a 5% non-response rate. A total of 285 HVs were allocated to Addis Ababa, and 154 HVs were selected for PM_2.5_ measurements. Most studies recommend measuring PM_2.5_ levels in an area or city with between 20 and 100 HVs, with various types of venues, such as bars, hotels, restaurants, cafés, and nightclubs [19].

### 2.3. Sampling Techniques

The following six-sub cities in Addis Ababa were selected for this study based on the high number of registered HVs: Addis Ketema, Arada, Bole, Kirkos, Lideta, and Nefas-Silk-Lafto. Within each sub-city, the woreda with the highest number of HVs was chosen in consultation with the Tobacco Control Law Enforcement Team of the Addis Ababa Food, Medicine, and Healthcare Administration and Control Authority (FMHACA). The list of HVs at the sub-city’s Bureau of Trade and the Woreda Office was incomplete and inconsistent, so the selection process involved dividing the woreda into clusters and selecting 4–6 clusters with a high density of HVs and multiple streets, following the WHO’s recommended approach for this type of study [20].

We listed the number and types of HVs in the selected clusters by walking through each neighborhood. After compiling the names and categorizing the HVs in the delineated clusters, we allocated a sample size proportional to the various types of HVs (restaurants, cafés, hotels, grocery, bars, and nightclubs/lounges). A systematic random sampling approach was employed to select a subsample of 154 HVs for the air quality measurements.

Data collection was carried out for 10 days using a standardized checklist that was informed by the ‘How-to-Guide for Conducting Compliance Studies’ for the smoke-free law [21] and the provisions of the Ethiopian Tobacco Control Proclamation (1112/2019) [11]. The checklist was translated into Amharic and back-translated into English to ensure consistency. Covert observation of compliance with smoke-free laws in all study HVs was conducted using three pairs of trained data collectors and one supervisor using Open Data Kit (ODK) (https://opendatakit.org)-enabled smartphones. Electronic data were uploaded to a local server at Addis Ababa University (AAU), Addis Ababa, Ethiopia. 

### 2.4. PM_2.5_ Measurement

At the beginning of each data collection period, the Dylos DC1700 (manufacture: Dylos Corporation, 2900 Adams Street, Unit C38, Riverside, CA 92504, USA) was turned on to start recording the outside PM_2.5_ for a minimum of 30 min once a day. The device was left in operation, and particle concentrations were continuously measured until the end of the collection period. The purpose of outside air sampling was to establish comparative data for indoor air samples obtained on the same day [19]. Following the measurement of outdoor air quality, the data collectors entered the selected HV and identified the central location within the venue to assess the indoor PM_2.5_. They placed the Dylos DC1700 equipment away from open doors, windows, mechanical ventilation, open flames, or other sources of SHS to minimize external interference. To ensure accurate indoor measurements, the bag containing the monitor was placed at table or chair level at least 1 m from any smoker, and beverages were ordered for the data collectors. Indoor measurements were performed for a minimum of 30 min.

After each period, the Dylos DC1700 was turned off, and the data were downloaded to a PC using Dylos Logger software version 3. The start and end times for each venue’s PM_2.5_ recording were also recorded, along with the type of venue, entrance and exit times, room size, number of people present, number of people using tobacco products, presence of mechanical ventilation, presence of any source of smoke, and whether doors/windows were open. The peak hours for HVs were considered to be between 18:00 and 24:00 h East Africa Time (EAT) for observation and measurement of PM_2.5_ levels.

### 2.5. Data Analysis

Data cleaning and analysis were performed using SPSS version 26 and STATA version 14. Descriptive statistics, such as frequencies, proportions, mean, median, interquartile range (IQR), and standard deviations (SDs), were used to summarize the data. Seven specific smoke-free law indicators were used to assess indoor space compliance, including the absence of smoking, ashtrays, lighters, shisha equipment, and tobacco products within 10 m of any door, window, or air intake mechanism. 

For each HV type, the overall average compliance with the smoke-free law was determined using a method employed in previous studies [16,21,22], where the compliance percentages for each smoke-free law indicator were added and the sum was divided by the total number of indicators. Differences between groups were assessed using a t-test, with the level of statistical significance set at *p* < 0.05 for.

The Dylos DC1700 measures particle number concentrations up to 0.5 µm (small particles) and 2.5 µm and above (large particles) in 0.01 cubic foot of air for each minute of measurement. The objective of this study was to determine the particle number concentrations between large and small particles, and the large-particle reading was subtracted from the small-particle reading. These values were multiplied by 100 to obtain the number of particles per cubic foot of air. Finally, the particle number concentrations were converted to PM_2.5_, using a formula proposed in previous studies [5,16].

In addition to the indoor PM_2.5_ measurements, 26 outside air measurements were conducted to provide comparative data; however, one measurement was incomplete and excluded from the analysis. PM_2.5_ data were analyzed using MS Excel and SPSS version 24. The average PM_2.5_ concentration data for each minute’s number of particle concentrations reported by the Dylos DC1700 was generated for each HV. The means and medians of the average PM_2.5_ concentration were compared based on venue type, size of the venue, compliance status (compliant vs. non-compliant), active tobacco smoke, and other sources of smoke. Statistical significance was set at *p* < 0.05. Additionally, the indoor PM_2.5_ concentrations (µg/m^3^) in the current study were compared with the standard air quality index breakpoints over a 24 h average. The WHO five air quality categories are good (0.0–15.0 µg/m^3^), moderate (15.1–40.0 µg/m^3^), unhealthy for sensitive groups (40.1–65.0 µg/m^3^), unhealthy for all (65.1–250 µg/m^3^), and hazardous (>250 µg/m^3^) [6]. For each type of venue, the percentage (%) greater than the average WHO 24 h level (15 µg/m^3^) was calculated with a 95% confidence interval (CI). As a checklist for the submission of this manuscript, we used STROBE cross-sectional reporting guidelines [23].

## 3. Results

### 3.1. General Characteristics of the HVs

Data from only 144 (93.5%) of the 154 HVs were used for the final analysis, as four venues were measured for less than 30 min, and six were measured incorrectly. Most of the HVs included in this study were selected from Bole sub-city, Woreda 03 (n = 38, 26%); Nifas-Silk-Lafto sub-city, Woreda 01 (n = 26, 18.1%); and Kirkos sub-city, Woreda 02 (n = 24, 16.7%) (Table 1). Approximately 27% (n = 39) were bars and restaurants, 17.4% (n = 25) were bars, and 15.3% (n = 22) were nightclubs. Most venues (88.9%, n = 128) had only indoor facilities, whereas 11.1% (n = 16) had both indoor and outdoor facilities. Considering the potential accommodation size of the venues, 42.4% (n = 61) were categorized as large (>45 persons), 34.7% (n = 50) as medium (30–45 persons), and the remaining 22.9% (n = 33) as small (<30 persons) (Table 1).

**Figure 1:**
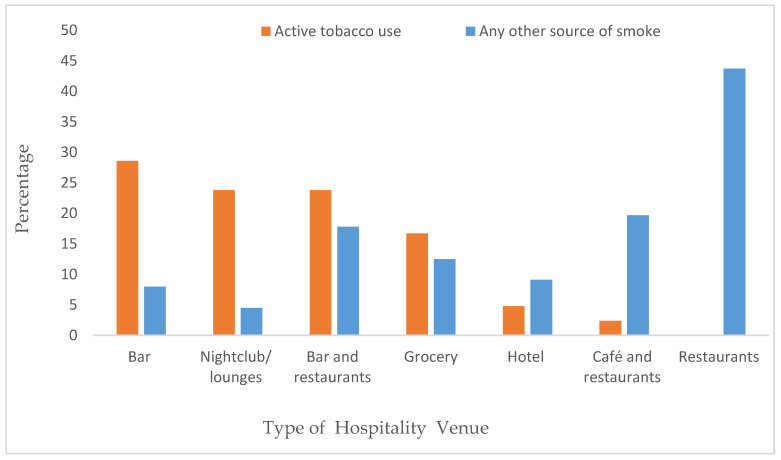
Source of smoke in hospitality venues in Addis Ababa.

Active tobacco smoking was observed in various HVs, with the highest prevalence observed in bars (28.6%) followed by nightclubs/lounges (23.8%). No active tobacco use was observed in restaurants. Additionally, at least one other source of smoke, such as an open kitchen, coal, or candle, was present in 43.7% of restaurants and 19.7% of cafés and restaurants (Figure 1).

### 3.2. PM_2.5_ Concentrations in Indoor HVs

The overall mean and median PM_2.5_ concentrations for indoor measurements were 37.23 µg/m^3^ and 18.92 µg/m^3^ (IQR: 23.26), respectively, and those for outside measurements were 15.89 µg/m^3^ and 14.53 µg/m^3^ (IQR: 8.44), respectively. The outside PM_2.5_ concentrations (15.92 µg/m^3^) in our study were almost equivalent to the WHO’s 24 h average PM_2.5_ level (15 µg/m^3^). Table 2 shows the PM_2.5_ levels according to type of HV, nature, and size of the venue. The median concentrations of PM_2.5_ were highest in restaurants (21.91 µg/m3, IQR: 29.45), bars (21.61 µg/m^3^, IQR: 28.85), and grocery stores (21.39 µg/m^3^, IQR: 11.43). Interestingly, the median PM_2.5_ level in nightclubs/lounges (12.12 µg/m^3^, IQR: 48.07) was lower than in the other HV categories, despite their mean PM_2.5_ level (45.28 µg/m^3^) being higher, except for restaurants, which had a mean of 60.4 µg/m^3^. The median PM_2.5_ concentrations were similar across the three sizes of HVs.

### 3.3. Compliance with Smoke-Free Law in HVs

**Figure 2:**
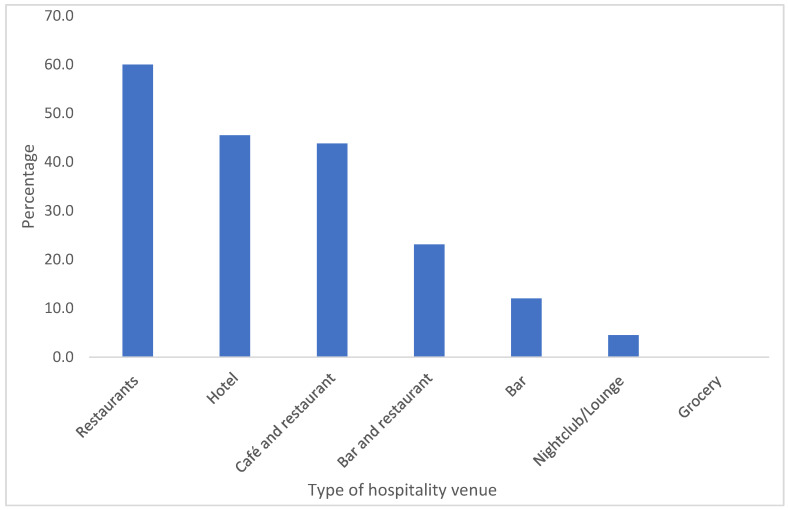
Indicators of compliance with smoke-free laws by type of hospitality venue in Addis Ababa.

Average compliance to smoke free laws indicators in HVs, restaurants (60%), hotel (45.5%) and café and restaurants (43.8%) had the highest proportion, while grocery, nightclubs/lounges, and bars showed the lowest compliance rates, at 0%, 4.5%, and 12%, respectively. Across all HVs, only 23.6% demonstrated full compliance with smoke-free laws (Figure 2).

The percentage of HVs with indoor PM_2.5_ concentrations (µg/m^3^) exceeding the WHO’s 24 h average (>15 µg/m^3^) and their respective 95% CIs. Overall, 63.9% (95% CI: 56–72%) of HVs had PM_2.5_ concentrations greater than 15 µg/m^3^. Grocery (81.3%) had the highest proportion, followed by cafés and restaurants (73.3%), bars and restaurants (69.2%), and restaurants (68.8%). The 95% CIs overlapped, suggesting that there were no significant differences. The proportion of HVs with indoor PM_2.5_ concentrations greater than the WHO’s 24 h average was 75% in venues with both indoor and outdoor facilities, compared to 62.5% in venues with only indoor facilities, with no statistically significant difference observed. In terms of venue size, similar percentages (62.0–66.7%) of the three venue sizes surpassed the WHO’s 24 h average PM_2.5_ (µg/m^3^) (Table 3).

Table 4 indicates that 36.1% (n = 52) of HVs had PM_2.5_ concentrations below 15 µg/m^3^, which is considered good, while 40.3% (n = 58) had PM_2.5_ concentrations between 15.1 and 40 µg/m^3^, categorized as moderate. Approximately 11% (n = 16) and 10.4% (n = 15) of the venues had PM_2.5_, which is considered unhealthy for sensitive groups and all people, respectively. The air quality in two restaurants and one nightclub/lounge was deemed hazardous. Furthermore, 11.7% (n = 15) of HVs with only indoor facilities, 16% (n = 4) of bars, and two nightclubs/lounges had unhealthy air quality levels.

**Figure 3:**
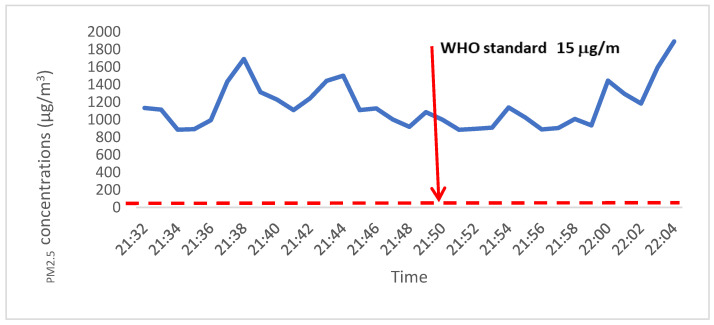
PM_2.5_ (µg/m^3^) measurement at a nightclub with several tobacco users (Addis Ababa, December 2022).

Figure 3 shows a PM_2.5_ concentration (µg/m^3^) measurement in one of the nightclubs/lounges where several people were using tobacco products. This HV recorded a hazardous level of PM_2.5_ concentration (>250 µg/m^3^) at the time of indoor data collection.

Table 5 shows the PM_2.5_ concentrations (µg/m^3^) inside the HVs based on compliance with smoke-free laws. Venues where smoking was observed had a median PM_2.5_ concentration of 22.56 µg/m^3^ (IQR: 48.41), which is greater than the median concentration of 17.06 µg/m^3^ (IQR: 17.54) in venues without smoking. Although the difference was not statistically significant (*p* = 0.099), smoking venues had higher median PM_2.5_ values. The presence of multiple smokers, shisha equipment, and the sale of tobacco products in indoor spaces were all significantly associated with higher PM_2.5_ concentrations (*p* < 0.005). Although open kitchens and coal smoke contributed to an increase in PM_2.5_, none of these associations was statistically significant.

## 4. Discussion

This study is the first to be conducted in Ethiopia in accordance with smoke-free laws that measures PM_2.5_ concentration in HVs. The mean and median PM_2.5_ concentrations for indoor measurements were 37.23 µg/m^3^ and 18.92 µg/m^3^, respectively, compared to outdoor measurements of 15.89 µg/m^3^ and 14.53 µg/m^3^, respectively. Approximately 36% of HVs had PM_2.5_ concentrations below 15 µg/m^3^, while 40.3% had concentrations between 15.1 µg/m^3^ and 40 µg/m^3^. Approximately 11% and 10.4% of the venues had PM_2.5_ values considered unhealthy for sensitive groups and unhealthy for all people, respectively. Two restaurants and one nightclub had PM_2.5_ levels above 250 µg/m^3^, posing a health risk to both employees and customers in these establishments. Evidence of tobacco use in the indoor space of HVs was significantly associated with higher median PM_2.5_ concentrations (*p* < 0.005).

The average outdoor PM_2.5_ concentration was comparable to the WHO’s 24 h average outdoor measurement (15 µg/m^3^) [6], but it was significantly lower than the indoor measurements, indicating potential PM_2.5_ emissions in indoor HVs. Previous research in different settings has also reported higher indoor PM_2.5_ concentrations than outdoor measurements [18,24]. In our study, the presence of multiple smokers and shisha equipment, as well as the sale of tobacco products in indoor spaces, was significantly associated with a higher median PM_2.5_. We also found hazardous levels of PM_2.5_ in a nightclub/lounge during new year celebrations, where several people were smoking tobacco products. Our findings align with a study conducted in Ghana [25] that reported higher PM_2.5_ concentrations (28.3 μg/m^3^) in HVs with indoor tobacco smoking compared to smoke-free spaces (12.3 μg/m^3^). Consistent with other studies, our research demonstrates an increase in PM_2.5_ concentrations in the presence of tobacco smoke [9,25,26].

In this study, 63.9% of HVs had indoor PM_2.5_ concentrations (µg/m^3^) higher than the WHO’s 24 h average (>15 µg/m^3^) [6]. These estimates are notably high in countries with comprehensive smoke-free laws. However, no studies have been conducted in Ethiopia before or after the approval of smoke-free laws, and we lack a reference with which to compare our findings. A study conducted in Turkey reported that cigarette smoking was observed in 67.5% of HVs, with a median PM_2.5_ five times higher than ours, even after the implementation of smoke-free laws [3]. Conversely, studies conducted in Michigan [27] and Scotland [9] reported a significant decline in active indoor smoking and PM_2.5_ concentration following the implementation of such laws. This suggests a potential impact of strong regulatory measures in reducing SHS exposure.

Although no active tobacco smoking was observed in restaurants, 68.8% had PM_2.5_ concentrations exceeding 15 µg/m^3^. This can be explained by PM_2.5_ emissions resulting from biomass fuel combustion, fuel combustion for heating, vehicular traffic, and other human activities [28,29]. Studies conducted in rural Malawi, Ethiopia, and Uganda [28,29,30] also have reported similar results. 

Approximately 24% of HVs complied with all smoke-free law indicators, although cigarette smoking (29.2%) and shisha smoking (6.2%) were observed. Notably, bars and nightclubs/lounges had the lowest smoke-free compliance rates, and active tobacco use was observed. This finding aligns with previous studies that reported higher tobacco use and poorer compliance with smoke-free laws in bars and pubs than in hotels and other HVs [13,30]. Ethiopia has strong tobacco control laws, as evidenced by the comprehensive tobacco control law enacted in 2019 and reinforced by the Tobacco Control Directive (Number 771/2021) of 2021 [26]. The Proclamation mandates that indoor public spaces, public transport, and workplaces be completely free of smoke [11]. However, our study reveals suboptimal implementation of these laws in HVs, particularly in bars and nightclubs/lounges where law enforcement activities are limited.

## 5. Limitations

The PM_2.5_ concentration was measured over a 30 min period, while the WHO’s air quality standard employs data from a 24 h measurement. This difference in duration might affect comparability of the results. However, the results of this study indicate that the measured average outdoor PM_2.5_ concentration was comparable to the WHO’s 24 h average outdoor measurement. This increases our confidence that the higher average indoor PM_2.5_ concentration measurement was due to a potential source of PM_2.5_ emissions in indoor HV environments. Although tobacco is the main source of PM_2.5_ emissions, biomass fuels, fuel combustion for heating, vehicles, and similar human activities can also be sources [5,28,29,30].

## 6. Conclusions

Compliance of HVs in Addis Ababa with smoke-free law indicators was found to be suboptimal. Active tobacco smoking is more common in bars and nightclubs than other types of HVs. In 64% of HVs, PM_2.5_ concentrations exceeded the average WHO air quality standard. Active use of cigarettes and shisha contributes to elevated PM_2.5_ emissions in HVs. It is crucial to cautiously interpret the high PM_2.5_ concentrations in smoke-free HVs, surpassing the WHO air quality standard, considering other potential sources of PM_2.5_. Further research and interventions are needed to address additional contributors to PM_2.5_ levels in HVs in Addis Ababa. It is recommended that the enforcement of smoke-free laws be strengthened, particularly for bars and nightclubs/lounges. We recommend that similar studies be conducted in other regions of Ethiopia.

## Figures and Tables

**Table 1:** Characteristics of hospitality venues by sub-city and woreda.

	Name of Sub-City						Total
	Addis Ketema n (%)	Arada n (%)	Bole n (%)	Lideta n (%)	Kirkos n (%)	Nifas-Silk Lafto n (%)	n (%)
**Type of venue**							
Restaurant	2 (14.3)	1 (5.0)	4 (10.5)	3 (13.6)	3 (12.5)	3 (11.5)	16 (11.1)
Café and restaurant	2 (14.3)	3 (15.0)	3 (7.9)	4 (18.2)	2 (3.8)	1 (3.8)	15 (10.4)
**Bar and restaurant**	3 (21.4)	5 (25.0)	12 (31.6)	5 (22.7)	5 (20.8)	9 (34.6)	39 (27.1)
Hotel	2 (14.3)	1 (5.0)	4 (10.5)	1 (4.5)	2 (8.3)	1 (3.8)	11 (7.6)
Grocery	3 (21.4)	1 (5.0)	4 (10.5)	4 (18.2)	2 (8.3)	2 (7.7)	16 (11.1)
Bar	2 (14.3)	4 (20.0)	4 (10.5)	3 (13.6)	5 (20.8)	7 (26.9)	25 (17.4)
Nightclub/lounge	0.0	5 (25.0)	7 (18.4)	2 (9.1)	5 (20.8)	3 (11.5)	22 (15.3)
**Nature of venue**							
Indoor and outdoor facility	2 (14.3)	1 (5.0)	1 (2.6)	1 (4.5)	1 (4.2)	10 (38.5)	16 (11.1)
Only indoor facility	12 (85.7)	19 (95.0)	37 (97.4)	21 (95.5)	23 (95.8)	16 (61.5)	128 (88.9)
**Venue size**							
Small (<30 persons)	6 (42.9)	3 (15.0)	9 (23.7)	9 (40.9)	3 (12.5)	3 (11.5)	33 (22.9)
Medium (30–45 persons)	6 (42.9)	12 (60.0)	4 (10.5)	7 (31.8)	9 (37.5)	12 (46.2)	50 (34.7)
Large (>45 persons)	2 (14.3)	5 (25.0)	25 (65.8)	6 (27.3)	12 (50.0)	11 (42.3)	61 (42.4)
Total, n (%)	14 (9.7)	20 (13.9)	38 (26.4)	22 (15.3)	24 (16.7)	26 (18.1)	144 (100.0)
Outside PM_2.5_ measurement	3 (12.0)	4 (16.0)	6 (24.0)	5 (20.0)	3 (12.0)	4 (16.0)	25 (100.0)

**Table 2:** Indoor PM_2.5_ concentrations (µg/m^3^) by the type and size of hospitality venue.

Type and Nature of Venue	n (%)	Mean	Median	IQR *	Min	Max	Median *p*-Value **
Restaurant	16 (11.1)	60.40	21.91	29.45	6.99	338.55	0.307
Café and restaurant	15 (10.4)	26.84	17.46	16.50	8.62	121.93	
Bar and restaurant	39 (27.1)	38.57	19.27	36.80	6.19	164.10	
Hotel	11 (7.6)	20.46	15.48	17.77	8.73	56.97	
Grocery	16 (11.1)	26.70	21.39	11.43	10.63	84.60	
Bar	25 (17.4)	33.50	21.61	28.85	4.93	197.84	
Nightclub/lounge	22 (15.3)	45.28	12.12	48.07	4.88	258.84	
**Nature of the venue**							
Indoor and outdoor facility	16 (11.1)	28.36	23.90	26.39	6.00	63.56	0.426
Only indoor facility	128 (88.9)	38.35	17.94	23.76	4.88	338.55	
**Venue size**							
Small (<30 persons)	33 (22.9)	36.02	19.96	36.76	8.62	258.84	0.780
Medium (30–45 persons)	50 (34.7)	32.42	17.05	18.75	4.88	197.84	
Large (>45 persons)	61 (42.4)	41.79	19.27	25.11	5.06	338.55	
Indoor PM_2.5_ concentration	144 (100)	37.23	18.92	23.09	4.88	338.55	
Outdoor PM_2.5_ concentration	25	15.92	14.20	8.97	5.08	41.89	

* IQR= interquartile range; ** significance of 0.05.

**Table 3:** Comparison of indoor PM_2.5_ concentrations (µg/m^3^) of the venues with the WHO’s 24 h average.

Type and Nature of Venue	Number	Percentage (%) Greater Than the WHO’s 24 h Average Level (15 µg/m^3^), (95% CI)
Restaurant	11	68.8 (43–94)
Café and restaurant	11	73.3 (48–99)
Bar and restaurant	27	69.2 (54–84)
Hotel	6	54.5 (19–90)
Grocery	13	81.3 (60–99)
Bar	16	64.0 (44–84)
Nightclub/lounge	8	36.4 (15–58)
**Nature of the venue**		
Both indoor and outdoor facility	12	75.0 (51–99)
Only indoor facility	80	62.5 (54–71)
**Venue size**		
Small (<30 persons)	22	66.7 (50–84)
Medium (30–45 persons)	31	62.0 (48–76)
Large (>45 persons)	39	63.9 (52–76)
Total (%)	92	63.9 (56–72)

**Table 4:** Comparison of indoor PM_2.5_ concentrations (µg/m^3^) of the venues with standard air quality index breakpoints (Addis Ababa, December 2022).

Type and Nature of Venue, n (%)	Air Quality Breakpoints (µg/m^3^, 24-h Average)				
	Good (0.0–15.0)	Moderate (15.1–40.0)	Unhealthy for Sensitive Groups (40.1–65.0)	Unhealthy for All (65.1–250)	Hazardous (>250)
Restaurant	5 (31.3)	7 (43.8)	2 (12.5)	0.0	2 (12.5)
Café and restaurant	4 (26.7)	8 (53.3)	2 (13.3)	1 (6.7)	0.0
Bar and restaurant	12 (30.8)	15 (38.5)	7 (17.9)	5 (12.8)	0.0
Hotel	5 (45.5)	5 (45.5)	1 (9.0)	0.0	0.0
Grocery	3 (18.8)	11 (68.8)	1 (6.3)	1 (6.3)	0.0
Bar	9 (36.0)	10 (40.0)	2 (8.0)	4 (16.0)	0.0
Nightclub/lounge	14 (63.6)	2 (9.1)	1 (4.5)	4 (18.2)	1 (4.5)
**Nature of the venue**					
Both indoor and outdoor facility	4 (25.0)	8 (50.0)	4 (25.0)	0.0	0.0
Only indoor facility	48 (37.5)	50 (39.1)	12 (9.4)	15 (11.7)	3 (2.3)
**Venue size**					
Small (<30 persons)	11 (33.3)	12 (36.4)	5 (15.2)	4 (12.1)	1 (3.0)
Medium (30–45 persons)	19 (38.0)	21 (42.0)	5 (10.0)	5 (10.0)	0.0
Large (>45 persons)	22 (36.1)	25 (41.0)	6 (9.8)	6 (9.8)	2 (3.3)
Total, n (%)	52 (36.1)	58 (40.3)	16 (11.1)	15 (10.4)	3 (2.1)

**Table 5:** Indoor PM_2.5_ concentrations (µg/m^3^) by smoke-free law indicators and other sources of smoking (Addis Ababa, December 2022).

	N (%)	Mean	Median	IQR *	Min	Max	Median *p*-Value **
Cigarette smoking in the indoor space							
Yes	42 (29.2)	46.17	22.56	48.41	4.88	258.84	0.099
No	102 (70.8)	33.53	17.06	17.54	4.93	338.55	
Number of people smoking cigarettes							
None	102 (70.8)	34.31	17.06	19.99	4.88	338.55	0.001 **
One	19 (13.2)	21.73	14.93	13.23	6.55	66.40	
Two	8 (5.6)	53.17	21.20	34.44	5.68	258.84	
Three or more	15 (10.4)	68.11	49.94	62.99	8.41	246.06	
shishas observed							0.02 **
Yes	5 (3.5)	139.8	96.4	233.8	32.6	348.04	
No	139 (96.5)	28.5	16.7	17.26	4.7	318.5	
‘No smoking sign’ posted in the indoor space							0.884
Yes	85 (59.03)	33.9	17.3	17.36	4.95	348.04	
No	59 (40.97)	30.2	15.5	21.24	4.7	247.9	
Smoking tobacco within 10 m from any air intake mechanism							
Yes	83	30.3	15.8	14.28	4.7	348	0.244
No	61	35.26	17.35	19.3	5.66	318.47	
Sale of tobacco products in the indoor space							
Yes	3 (2.08)	88.8	58.4	-	20.89	187.13	0.043 **
No	141 (99.92)	31.2	16.8	18.12	4.7	348.04	
Door/window opened (n = 143)							
Yes	127 (88.8)	34.12	18.42	22.20	5.06	338.55	0.768
No	16 (11.2)	62.63	21.81	65.98	4.88	258.84	
Open kitchen							
Yes	6 (4.2)	69.49	48.27	113.18	17.46	164.10	0.677
No	138 (95.8)	35.81	18.17	21.59	4.88	338.55	
Presence of coal smoke							
Yes	8 (5.6)	76.32	32.99	63.9	14.54	338.55	0.275
No	136 (94.4)	34.92	18.06	22.79	4.88	295.35	
Total, n (%)	144	37.22	18.92	23.26	4.88	33.55	

* IQR = interquartile range; ** significance level of 0.05.

## Data Availability

The datasets used and analyzed in the current study will be made available by the corresponding author upon reasonable request.

## Operational Definitions

Active smoking	Possession or control of a lit tobacco product, including cigarettes, cigars, and shisha, inside or outside HVs at the time of data collection.
Hospitality venue	An establishment registered under the regulation of the Government of Ethiopia where food and beverages are sold and consumed, namely hotels, restaurants, bars, bars and restaurants, cafés and restaurants, butcher houses and restaurants, grocery, nightclubs, and lounges.
Bar	An establishment where alcoholic drinks and (sometimes) food are served to clients.
Café	An establishment where simple meals and drinks (such as tea, coffee, and milk) are served to clients.
Compliance	The degree to which HVs fully implement tobacco control laws under the 2019 Ethiopian Tobacco Control Law (Proclamation No. 1112/2019).
Grocery	A small pub where individuals buy and drink alcoholic beverages. Most of these establishments are informal, neighborhood-based, and serve a variety of local and commercial alcoholic drinks.
Hotel	An establishment offering lodging, food, beverages, as well as recreational, conference, and similar facilities, to clients.
Lounge	A place where customers enjoy alcoholic beverages while listening to soothing music or watching television. In this study, however, lounges serve as nightclubs, since the latter is regarded as illegal.
Nightclub	A place of entertainment open to clients at night, usually serving food and liquor and providing music and space for dancing.
Restaurant	An establishment offering food and beverage services to clients.
Tobacco product	A product entirely or partly made of tobacco leaf as a raw material that is manufactured for use in smoking, chewing, sucking, or snuffing.

## List of Abbreviations

AAU	Addis Ababa University
DSA	Designated smoking area
EAT	East Africa Time
FCTC	Framework Convention on Tobacco Control
GATS	Global Adult Tobacco Survey
HVs	Hospitality Venues
IQR	Interquartile Range
ODK	Open Data Kit
SD	Standard Deviation
SF	Smoke-free
SHS	Second-hand Smoke
PM	Particulate Matter
WHO	World Health Organization
